# Impact of Nutritional Literacy and Food Label Reading Habits on Healthy Lifestyle Behaviors Among Pregnant Women in Türkiye: A Cross‐Sectional Study

**DOI:** 10.1002/fsn3.71797

**Published:** 2026-04-17

**Authors:** Mehmet Arif Icer, İrem İlayda Sezen, Zehra Tuncay, Aybike Gizem Köse, Ozge Yesildemir

**Affiliations:** ^1^ Department of Nutrition and Dietetics, Faculty of Health Sciences Amasya University Amasya Türkiye; ^2^ Department of Nutrition and Dietetics, Faculty of Health Sciences Bursa Uludag University Bursa Türkiye

**Keywords:** food labels reading, healthy lifestyle behaviors, nutrition literacy, pregnancy, pregnant women

## Abstract

The number of studies examining the effects of nutritional literacy and food label reading habits on the healthy lifestyle behaviors of pregnant women is quite limited. This study aimed to assess the levels of food label reading and nutrition literacy of pregnant women in Türkiye and to examine their effects on healthy life behaviors. The current study is cross‐sectional and was conducted on a total of 370 pregnant women. All participants were asked to provide descriptive information, nutritional habits, and details about their pregnancies. The Healthy Lifestyle Behaviors in Pregnancy Scale (HLBPS), the Food Label Reading Attitude Scale (FLRAS), and the Food and Nutrition Literacy Scale (FNLS) were administered to the participants. The study found that as FLRAS and FNLS scores increased, HLBPS scores also increased (*r* = 0.21, *p* < 0.001; *r* = 0.15 *p* < 0.01, respectively). Regression analysis showed that food label reading attitude (*B* = 0.15, 95% CI: 0.05–0.25, *β* = 0.15, *p* < 0.05) and food and nutrition literacy (*B* = 0.29, 95% CI: 0.15–0.43, *β* = 0.21, *p* < 0.001) were significant predictors of healthy lifestyle behaviors. When both variables were entered simultaneously, food and nutrition literacy remained a significant predictor (*B* = 0.26, 95% CI: 0.12–0.40, *β* = 0.19, *p* < 0.001). Moreover, according to HLBPS scores, it was seen that the FLRAS scores of the participants in the first tertile were lower than the participants in the second and third tertiles (*p* < 0.05), while the FNLS scores of the participants in the third tertile according to HLBPS scores were higher than the participants in the first and second tertiles (*p* < 0.05). These findings underscore the importance of integrating nutrition literacy and label‐use strategies into prenatal care services, which may help improve healthy lifestyle behaviors among pregnant women, in line with recent recommendations in the literature.

## Graphical abstract

This study of 370 pregnant women in Türkiye found that higher food and nutrition literacy and a positive attitude toward reading food labels significantly predict healthier lifestyle behaviors during pregnancy. Regression analyses confirmed that both factors are key contributors, even after adjusting for socioeconomic variables. The findings highlight the need to integrate nutrition education and label‐reading guidance into routine prenatal care to improve maternal and neonatal health outcomes.

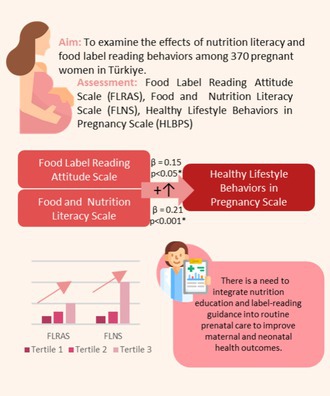

## 1 Introduction

Maternal and child health is a critical global health indicator. In middle‐income countries, the focus has gradually shifted from a historical emphasis on reducing mortality to a broader approach that includes improving overall maternal and infant health outcomes (Li and Wang 2025). Central to this shift is the recognition of healthy maternal nutrition during pregnancy as a key determinant of both maternal and neonatal well‐being (Aktaç et al. 2024; Sibai et al. 2022; Slater et al. 2020). Pregnant women are generally more receptive to health advice and are more likely to plan their diet during pregnancy, as they become increasingly aware of the impact of nutrition on fetal health (Bookari et al. 2016b).

Emerging evidence indicates that nutritional knowledge alone is insufficient to achieve sustained behavior change among pregnant women (Li and Wang 2025; Melwani et al. 2022; Uzan et al. 2024). The concept of nutrition literacy—an extension of health literacy within the context of nutrition—addresses this gap by encompassing not only knowledge, but also the ability to access, understand, critically evaluate, and apply nutritional information in everyday life. Low levels of nutrition literacy have been identified as a significant barrier to healthy dietary behaviors, particularly among pregnant women, who often face conflicting information, limited autonomy in decision‐making, and strong familial or cultural influences (Al‐Mutawtah et al. 2023; Grenier et al. 2021; Li and Wang 2025). Nutritional literacy, a key factor influencing food choices, consumption, and purchasing behaviors, plays a critical role in the development of healthy eating habits (Bomfim et al. 2020; Jones and Adkins 2021; Koca and Arkan 2021). Studies have shown that higher nutritional literacy is associated with better dietary choices and better health outcomes (Koca and Arkan 2021; Qi et al. 2023). However, there is also a study in the literature that shows that there is no relationship between nutritional literacy level and eating habits (Taleb and Itani 2021). Beyond the general population, the potential effects of nutritional literacy on healthy lifestyle behaviors—such as pregnancy responsibility, nutrition, and physical activity—are also an important area of research in pregnant women (Nguyen et al. 2025; Papežová et al. 2023). Nguyen et al. (2025) reported that increasing the nutritional literacy levels of pregnant women is essential for improving the health of both mothers and their unborn babies and for fostering the development of healthy eating habits. It is believed that learning about self‐regulation and self‐control strategies can also contribute to dietary control and guide individuals toward healthy food choices (Diotaiuti et al. 2022).

Nutrition labels, which are an important communication tool between food manufacturers and consumers, encourage consumers to make healthier food choices (İçer and Karadağ 2023). A food label is any label, brand, sign, picture or other descriptive statement written, printed, stenciled or marked on a food package or container (Odaman et al. 2020). It is reported that educational interventions targeting improvements in food literacy, including nutritional knowledge, food label interpretation skills, and cooking skills, will promote healthy living (Mancone et al. 2024). Although food label usage habits have been studied among specific populations such as adolescents (Yilmazel and Bozdogan 2021) and university students (Yardımcı and Demirer 2022) in Türkiye, it is surprising that pregnant women have been overlooked. In their study, In their study, Kim et al. (2017) found no statistically significant difference in food label reading habits between pregnant and non‐pregnant women in the United States. Determining food label usage habits among pregnant women and examining the relationships of this habit with nutritional literacy and healthy lifestyle behaviors is important in terms of revealing how effective this factor is in maintaining healthy lifestyle behaviors during pregnancy.

The relationship between nutrition literacy, food label‐reading habits, and lifestyle behaviors represents an important area of research (Depboylu et al. 2023; Kempen et al. 2012). Depboylu et al. (2023) found that individuals who engage in higher levels of physical activity tend to have greater nutrition literacy compared to their less active counterparts. Similarly, Kempen et al. (2012) reported that frequent food label readers are more likely to adopt healthy lifestyle behaviors. These findings suggest that both nutrition literacy and label‐reading practices may contribute to improved health outcomes by promoting healthier lifestyle choices.

Current initiatives aimed at improving individual dietary habits emphasize the promotion of nutritional literacy, which includes the ability to access, understand, and use the essential nutrition information and services needed to make appropriate dietary decisions, as well as increasing food label reading habits (Nguyen et al. 2025). Recent interventions emphasize the role of food literacy in strengthening nutritional knowledge and self‐regulation. Yet, such multifaceted approaches have not been adequately tested among pregnant women. Addressing this gap is crucial, as pregnancy represents a unique window of opportunity to foster long‐term health behaviors that benefit both mother and child. To the best of our knowledge, there is no study conducted in Türkiye that simultaneously evaluates the impact of these two factors on the healthy lifestyle behaviors of pregnant women. In this context, the study seeks to address the following questions: What are the levels of food label reading attitudes and food and nutrition literacy among pregnant women in Türkiye? Is there a significant association between these factors and healthy lifestyle behaviors during pregnancy? And to what extent do food label reading attitudes and food and nutrition literacy predict healthy lifestyle behaviors among pregnant women? Therefore, the present study aims to address this gap by examining how food and nutrition literacy and food label reading attitudes relate to healthy lifestyle behaviors during pregnancy.

## 2 Materials and Methods

This cross‐sectional study was conducted on a total of 370 volunteer pregnant women aged between 18 and 42. The sample size was estimated as 354 pregnant women assuming 5% alpha error (i.e., confidence level = 95%) and 95% power. The software used for sample size calculation was G*Power 3.0.10. Experts in the field of Nutrition and Dietetics, pregnant women with chronic diseases, and/or on special diets have been excluded from the study. The study protocol was approved by the Ethics Committee of Amasya University (protocol code: 2024/127; date of approval: 26 November, 2024).

Participants were recruited from antenatal outpatient clinics of Amasya University Hospital and affiliated community health centers. The data for the study were collected using a face‐to‐face interview technique, during which participants provided self‐reported answers to structured questionnaires. The survey included questions about participants' demographic information (age, education level, occupation, income level, health status, pregnancy‐related information, smoking and alcohol use), as well as their eating habits, food label reading habits, and perceptions of food labels. In addition, the Healthy Lifestyle Behaviors in Pregnancy Scale (HLBPS), Food Label Reading Attitude Scale (FLRAS), and Food and Nutrition Literacy Scale (FNLS) were administered to the pregnant women.

### 2.1 Healthy Lifestyle Behaviors in Pregnancy Scale (HLBPS)

The Healthy Lifestyle Behaviors in Pregnancy Scale (HLBPS) was developed by Yılmaz and Karahan (2019), and its validity and reliability have been established. The scale consists of a total of 29 items and 6 subscales: hygiene, nutrition, physical activity, travel, accepting pregnancy, and pregnancy responsibility. Each subscale of the scale can be used independently in studies. This five‐point Likert scale is scored as follows: Never (1), Rarely (2), Sometimes (3), Often (4), and Always (5). The scale does not contain any reverse‐scored items. Higher scores on the scale indicate positive/high healthy lifestyle behaviors (Yılmaz and Karahan 2019). The internal consistency of the HLBPS was evaluated using Cronbach's alpha. In this present study, the Cronbach's alpha coefficient for the total scale was 0.72, indicating acceptable reliability.

### 2.2 Food Label Reading Attitude Scale (FLRAS)

To assess the participants' food label reading habits, the Food Label Reading Attitude Scale (FLRAS), developed by Sığırcı and Sarp (2024) and validated for reliability, was used. The survey is a five‐point Likert scale (Strongly Disagree = 1, Strongly Agree = 5) and consists of a total of 20 items. The minimum and maximum scores that individuals can obtain from the scale are 20 and 100, respectively (Yalçın and Sevim 2024). As the score on the scale increases, the attitude toward food label reading also improves (Sığırcı and Sarp 2024). The internal consistency of the FLRAS was evaluated using Cronbach's alpha. In this present study, the Cronbach's alpha coefficient for the total scale was 0.74, indicating acceptable reliability.

### 2.3 Food and Nutrition Literacy Scale (FNLS)

The Food and Nutrition Literacy Scale (FNLS) developed by Demir and Özer (2022) was used to assess participants' nutrition literacy. The scale consists of three dimensions: knowledge (0–13 points), attitude (13–65 points), and behavior (10–50 points). Higher scores on each subscale indicate better literacy in that domain. The total FNLS core ranges from 0 to 128, with higher scores reflecting greater overall food and nutrition literacy (Demir and Özer 2022).

### 2.4 Statistical Analysis

The research data were evaluated using the IBM SPSS Statistics for Windows version 27.0 (IBM Corp., Armonk, NY, USA) program. The mean, standard deviation, median, lower and upper values were calculated for the data obtained from the measurements of the individuals participating in the study. The distribution of qualitative data was given with number and percentage tables. Normality of continuous variables was assessed using Kolmogorov–Smirnov and Shapiro–Wilk tests. Cases with missing data (< 3%) were excluded from the analyses using listwise deletion. The *t*‐test was used to evaluate the means between two groups showing normal distribution, and the Mann–Whitney *U* test was used to evaluate the medians between two groups not showing normal distribution. The Kruskal‐Wallis Analysis of Variance was used to evaluate the median between three groups not showing normal distribution, and the Mann–Whitney *U* test was used for post hoc evaluation. The Pearson Correlation test was used to determine the strength and direction of the linear relationship between two continuous variables showing normal distribution, and the Spearman Correlation test was used between continuous variables not showing normal distribution. In this study, the HLBPS was analyzed using SPSS dividing the scores into tertiles. The tertile method was used to classify individuals into three different groups—low, medium, and high levels—by dividing the distribution of scale scores into three approximately equal parts. The tertile boundaries were determined based on the 33rd and 66th percentiles of the scale scores (≤ 98 = low; 99–112 = medium; ≥ 113 = high). This approach was chosen to facilitate group comparisons and interpretation of differences across levels of lifestyle behaviors. In contrast, FLRAS scores were analyzed as continuous variables since no validated cut‐off points or categorization methods have been established for this scale in the literature. Univariate linear regression analyses were conducted to examine the effects of Food Label Reading Attitude and Food and Nutrition Literacy on Healthy Lifestyle Behaviors during Pregnancy. Subsequently, multiple linear regression analysis was applied to assess the combined effects of these two variables. The statistical significance of the models was evaluated using the *F*‐test, and the contributions of the variables to the models were assessed through *B* coefficients, standardized *β* coefficients, and *p*‐values. Additionally, the explanatory power of the models was assessed using *R*‐squared (*R* ^2^) values. In Model 1, only Food Label Reading Attitude Scale was included, while Model 2 included only Food and Nutrition Literacy Scale. In Model 3, both independent variables were included in the model simultaneously, and Model 4 additionally included education, occupation and income as covariates. In all statistical tests, the confidence interval was accepted as 95.0% and *p* < 0.05 was considered significant.

## 3 Results

The sociodemographic and pregnancy‐related information of the participants, evaluated according to the HLBPS score tertiles, is presented in Table 1. The mean age of the participants in the first, second, and third tertile of the scale is 28.6 ± 5.2; 28.8 ± 4.8; and 28.9 ± 4.9 respectively (*p* > 0.05). The relationship between the educational status, occupation, income level of the participants, and healthy life behaviors is statistically significant (*p* < 0.05). It was observed that 29.2% of participants in the first tertile and 11.9% of participants in the third tertile have a household income level between 0–500 dollars. Additionally, 10.8% of participants in the first tertile and 29.4% of participants in the third tertile have a household income level between 1.500 and 3.000 dollars. A statistically significant relationship was found between the number of pregnancies and the age at which participants became pregnant and their HLBPS scores (*p* < 0.05). In all tertiles, most participants had one pregnancy, but the proportion of participants in the first tertile who had three or more pregnancies was higher. The age at which participants in the first tertile became pregnant for the first time (24.1 ± 4.0 years) is lower compared to the other participants, and this difference was found to be statistically significant (*p* < 0.05).

**TABLE 1:** Evaluation of pregnant women's sociodemographic and pregnancy‐related information according to their Healthy Lifestyle Behaviors in Pregnancy Scale scores (HLBPS).

Variables	Healthy lifestyle behaviors in pregnancy scale scores				*p*
	Total	Tertile 1	Tertile 2	Tertile 3	
Age (years) (mean ± SD)	28.7 ± 4.9	28.6 ± 5.2	28.8 ± 4.8	28.9 ± 4.9	*F* = 0.08 *p* > 0.05
Education level (*n* (%))					
Primary education	70 (18.9)	30 (25.0)	24 (19.4)	16 (12.7)	*X* ^2^ = 15.94 *p* < 0.001**
High school	139 (37.6)	53 (44.2)	43 (34.7)	41 (34.1)	
Bachelor's degree	153 (41.4)	36 (30.0)	55 (44.4)	62 (49.2)	
Master's degree/doctorate	8 (2.2)	1 (0.8)	2 (1.6)	5 (4.0)	
Occupation (*n* (%))					
Housewife	242 (65.4)	90 (75.0)	79 (63.7)	73 (57.9)	*X* ^2^ = 26.40 *p* < 0.05*
Government officer	57 (15.4)	10 (8.3)	17 (13.7)	30 (23.8)	
Worker	30 (8.1)	8 (6.7)	14 (11.3)	8 (6.3)	
Self‐employment	25 (6.8)	10 (8.3)	5 (4.0)	10 (7.9)	
Fee‐earning	8 (2.2)	—	6 (4.8)	2 (1.6)	
Student	8 (2.2)	2 (1.7)	3 (2.4)	3 (2.4)	
Level of income (dollar) (*n* (%))					
0–500 $	71 (19.2)	35 (29.2)	21 (16.9)	15 (11.9)	*X* ^2^ = 20.64 *p* < 0.001**
500–1.500 $	224 (60.5)	72 (60.0)	78 (62.9)	74 (58.7)	
1.500–3.000 $	75 (20.3)	13 (10.8)	25 (20.2)	37 (29.4)	
Number of pregnancies (*n* (%))					
One	159 (43.0)	42 (35.0)	61 (49.2)	56 (44.4)	*X* ^2^ = 9.97 *p* < 0.05*
Two	113 (30.5)	38 (31.7)	30 (24.2)	45 (35.7)	
> Three	98 (26.5)	40 (33.3)	33 (26.6)	25 (19.8)	
Current gestational week (*n* (%))					
First trimester (0–12 weeks)	53 (14.3)	16 (13.3)	20 (16.1)	17 (13.5)	*X* ^2^ = 4.00 *p* > 0.05
Second trimester (13–27 weeks)	107 (28.9)	42 (35.0)	34 (27.4)	31 (24.6)	
Third trimester (28 weeks and longer)	210 (56.8)	62 (51.7)	70 (56.5)	78 (61.9)	
Singleton pregnancy (*n* (%))					
Yes	361 (97.6)	118 (98.3)	119 (96.0)	124 (98.4)	*X* ^2^ = 2.01 *p* > 0.05
No	9 (2.4)	2 (1.7)	5 (4.0)	2 (1.6)	
Previous miscarriage (*n* (%))					
Yes	94 (25.4)	35 (29.2)	30 (24.2)	29 (23.0)	*X* ^2^ = 1.37 *p* > 0.05
No	276 (74.6)	85 (70.8)	94 (75.8)	97 (77.0)	
Age at first pregnancy (mean ± SD)	24.9 ± 4.3	24.1 ± 4.0^a^	25.5 ± 4.6^b^	25.2 ± 4.2^b^	*F* = 3.94 *p* < 0.05*
Total number of children (*n* (%))					
None	168 (45.4)	48 (40.0)	63 (50.8)	57 (45.2)	*X* ^2^ = 7.97 *p* > 0.05
One	117 (31.6)	36 (30.0)	34 (27.4)	47 (37.3)	
Two or more	85 (23.0)	36 (30.0)	27 (21.8)	22 (17.5)	
Smoking status (*n* (%))					
Never	296 (80.0)	93 (77.5)	105 (84.7)	98 (77.8)	*X* ^2^ = 5.75 *p* > 0.05
Quit smoking	8 (2.2)	4 (3.3)	3 (2.4)	1 (0.8)	
Quitting smoking during pregnancy	33 (8.9)	11 (9.2)	7 (5.6)	15 (11.9)	
Smoking	33 (8.9)	12 (10.0)	9 (7.3)	12 (9.5)	
Alcohol use status (*n* (%))					
Never	356 (96.2)	114 (95.0)	120 (96.8)	122 (96.8)	*X* ^2^ = 10.96 *p* > 0.05
Quitting alcohol use	3 (0.8)	—	—	3 (2.4)	
Quitting during pregnancy	10 (2.7)	5 (4.2)	4 (3.2)	1 (0.8)	
Alcohol use	1 (0.3)	1 (0.8)	—	—	

*Note:* ^a,b^Values shown with different letters are statistically significant. *p < 0.05, **p < 0.001.

Participants' HLBPS, FLRAS, and FNLS scores are given in Table 2. Participants' HLBPS mean score was 107.2 ± 10.8; FLRAS mean score was 81.0 ± 10.6; and FNLS mean score was 88.9 ± 7.6.

**TABLE 2:** Pregnant women's healthy lifestyle behaviors during pregnancy scale, food label reading attitude scale, and food and nutrition literacy scale scores.

Scales and sub‐dimensions	*X* ± SD	Median (Min–Max)
Healthy lifestyle behaviors in pregnancy scale	107.2 ± 10.8	107.0 (76.0–179.0)
Hygiene	19.5 ± 0.8	20.0 (18.0–20.0)
Nutrition	30.8 ± 5.1	31.0 (18.0–74.0)
Physical activity	9.0 ± 2.0	9.0 (4.0–15.0)
Travel	5.7 ± 2.3	5.5 ± (0.0–12.0)
Accepting pregnancy	5.9 ± 2.4	6.0 (0.0–15.0)
Pregnancy responsibility	19.3 ± 1.5	20.0 (13.0–20.0)
Food label reading attitude scale	81.0 ± 10.6	82.0 (38.0–100.0)
Food and nutrition literacy scale	88.9 ± 7.6	88.0 (64.0–112.0)
Knowledge domain dimensions	8.3 ± 3.1	8.0 (2.0–13.0)
Attitude domain dimensions	50.5 ± 3.8	50.0 (33.0–65.0)
Behavior domain dimensions	30.1 ± 3.9	30.0 (20.0–46.0)

**FIGURE 1:**
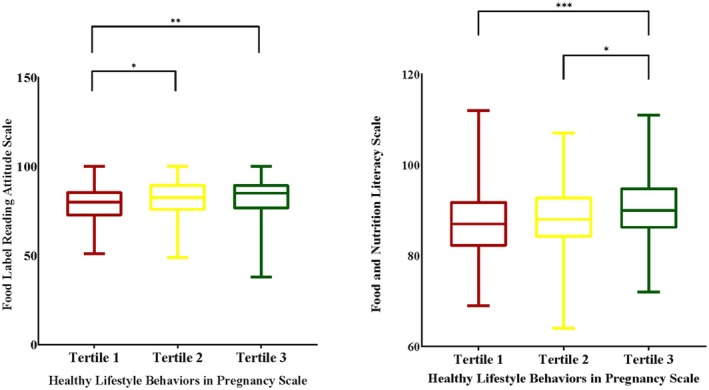
The relationship between tertiles of the healthy lifestyle behaviors in pregnancy scale and the food label reading attitude scale and food and nutrition literacy scale scores. *p < 0.05; **p < 0.005; ***p < 0.001.

The relationship between HLBPS tertiles and FLRAS and FNLS scores is illustrated in Figure 1. Statistically significant differences were found between HLBPS tertiles and participants' FLRAS mean scores (first, second, and third tertiles: 79.1, 81.7, and 82.2, respectively) (*p* < 0.05). Participants in the first tertile had lower FLRAS scores compared to those in the second and third tertiles (*p* < 0.05). Similarly, statistically significant differences were observed between HLBPS tertiles and participants' FNLS mean scores (first, second, and third tertiles: 87.0, 88.6, and 90.8, respectively) (*p* < 0.05). Participants in the third tertile had higher FNLS scores than those in the first and second tertiles (*p* < 0.05).

**FIGURE 2:**
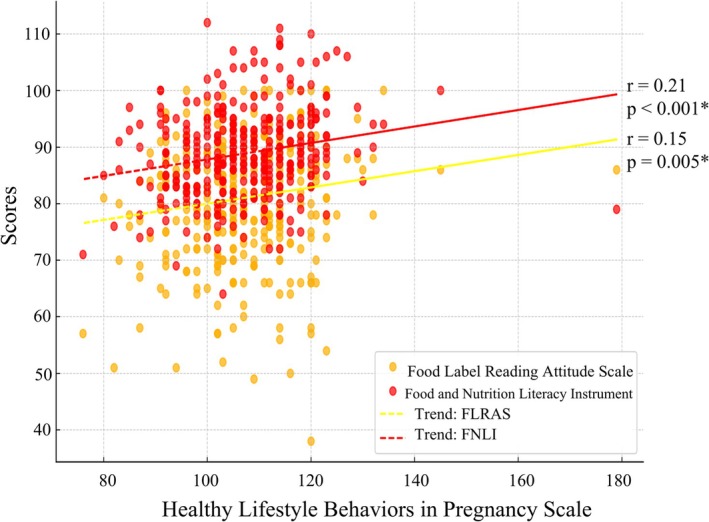
The relationship between the pregnant women's healthy lifestyle behaviors in pregnancy scale and the food label reading attitude scale and food and nutrition literacy scale scores.

The relationship between participants' HLBPS scores and FLRAS and FNLS scores is shown in Figure 2. Statistically significant positive correlations were found (*p* < 0.05), indicating that as FLRAS and FNLS scores increased, HLBPS scores also increased.

Table 3 presents the effects of food label reading attitudes and food and nutrition literacy on healthy living behaviors in pregnant women. A one‐point increase in FLRAS was associated with a 0.15‐point increase in the HLBPS score (*B* = 0.15, 95% CI: 0.05–0.25). Food label reading had a low but statistically significant effect on healthy living behaviors during pregnancy (*β* = 0.15; *p* < 0.05), explaining 2% of the variance in healthy living behaviors (*R* ^2^ = 0.02). A one‐point increase in FNLS was associated with a 0.29‐point increase in the HLBPS score (*B* = 0.29, 95% CI: 0.15–0.43). Food and nutrition literacy explained 4% of the variance in healthy living behaviors (*R* ^2^ = 0.04), with a moderate statistically significant effect (*β* = 0.21; *p* < 0.001). When both food label reading and food and nutrition literacy were considered together, a one‐point increase in these scales resulted in a 0.26‐point increase in the HLBPS score (*B* = 0.26). Together, they explained 6% of the variance in healthy living behaviors (*R* ^2^ = 0.06) and had a moderate significant effect (*β* = 0.19; *p* < 0.001). After including the covariates along the main predictor, food and nutrition literacy remained significant (*B* = 0.13, 95% CI: 0.001–0.32, *β* = 0.11; *p* < 0.05) while food label reading were marginally significant (*B* = 0.17, 95% CI: 0.01–0.22, *β* = 0.11; *p* = 0.05). The final model explained 8% of the total variance.

**TABLE 3:** Effect of food label reading attitudes and food and nutrition literacy on healthy lifestyle behaviors in pregnancy.

Variables	*B*	95% CI (Lower–Upper)	SE	*β*	*t*	*p*	*R* ^2^
Model 1							
Constant	95.06	86.59–103.53	4.31	—	22.01	< 0.05*	0.02
Food label reading	0.15	0.05–0.25	0.05	0.15	2.85		
Model 2							
Constant	81.36	68.71–94.00	6.43	—	12.65	< 0.001**	0.04
Food and nutrition literacy	0.29	0.15–0.43	0.07	0.21	4.04		
Model 3							
Constant	74.34	60.31–88.36	7.13	—	10.43	**<** 0.001**	0.06
Food Label Reading + Food and Nutrition Literacy	0.26	0.12–0.40	0.74	0.19	3.62		
Model 4							
Constant	82.36	67.39–97.34	7.61	—	10.82	< 0.001**	0.08
Food label reading	0.17	0.01–0.22	0.08	0.11	1.96	0.051	
Food and nutrition literacy	0.12	0.001–0.32	0.05	0.11	2.23	0.003*	

*Note:* Constant: Healthy Lifestyle Behaviors in Pregnancy Scale. *p < 0.05, **p < 0.001. Model 1: Food Label Reading Scale; Model 2: Food And Nutrition Literacy Scale; Model 3: Food Label Reading Scale + Food And Nutrition Literacy Scale; Model 4: Food Label Reading Scale + Food And Nutrition Literacy Scale + education status + occupation + income level.

## 4 Discussion

This study examines the levels of food label reading and nutrition literacy among pregnant women in Türkiye and their impact on healthy lifestyle behaviors. The results reveal that food label reading attitude and nutrition literacy positively influence healthy lifestyle behaviors in pregnant women. As food label reading and nutrition literacy increase, improvements in healthy lifestyle behaviors are observed.

When evaluating healthy lifestyle behaviors in detail, the HLBPS scores of our participants provide additional insights. Previous studies using similar methodologies have reported slightly higher total scores on this scale. For instance, the studies by Erkek and Altınayak (2024) (115.71 ± 15.80) and Baltacı et al. (2023) (113.99 ± 13.15) found mean scores that were approximately 8.5 and 6.8 points higher, respectively, compared to our findings. In addition, the distribution of HLBPS scores across tertiles suggests that pregnant women exhibited a relatively balanced proportion across lower, moderate, and higher adherence groups in the present study. This indicates that while some participants followed these behaviors more optimally, others demonstrated moderate or lower adherence. Overall, healthy lifestyle behaviors among pregnant women have been reported to be moderately prevalent (Jalili Bahabadi et al. 2020; Mihiret et al. 2025). Specifically, hygiene, nutrition, and physical activity scores were consistent with those reported in other studies, while notable differences were observed in the travel and pregnancy acceptance subscales (Baltacı et al. 2023; Erkek and Altınayak 2024). The travel score was significantly lower, possibly due to cultural norms, safety concerns, and limited transportation access. Similar findings have been reported in previous studies, where pregnant women's mobility and travel behaviors were influenced by cultural expectations, perceived safety risks, and infrastructural limitations (Roy et al. 2024; Mets‐Oja et al. 2025). Limited access to reliable transportation may restrict opportunities for prenatal care visits, physical activity, and social engagement, potentially impacting overall well‐being during pregnancy. Pregnancy acceptance was also lower, which may reflect emotional and psychological factors, such as social support and maternal mental well‐being. Low pregnancy acceptance has been associated with increased stress, anxiety, and adverse maternal–fetal outcomes in previous research (Moreau et al. 2022; Wu et al. 2024). These findings underscore the importance of considering both structural and psychosocial determinants when designing public health interventions to improve maternal health outcomes. Tailored programs that address cultural, logistical, and psychological barriers could enhance pregnant women's engagement in healthy lifestyle behaviors.

The behavior of pregnant women varies depending on their education level, age, number of pregnancies, sociocultural factors, personal characteristics, social and environmental influences, intrinsic and extrinsic motivation, and health knowledge (Papežová et al. 2023; Rockliffe et al. 2021). In this study, sociodemographic characteristics significantly influenced the HLBPS scores of participants. Education and income levels were positively associated with healthy lifestyle behaviors, suggesting that women with higher education and income are more likely to make informed health decisions. These findings align with another study showing that pregnant women with higher education and income tend to exhibit healthier lifestyle behaviors (Dalkılınç and Cetişli 2021). This may be because higher education enhances health literacy, enabling women to access, comprehend, and apply health‐related information more effectively. Supporting this, Rafat et al. (2025) indicated that higher educational attainment contributes to improved health knowledge. Higher income levels may also facilitate access to healthier food choices, prenatal care, and wellness resources, which support the adoption of healthy lifestyle behaviors. Additionally, our results showed that maternal age at first pregnancy and the number of pregnancies also played a role in shaping lifestyle behaviors. Women who had their first pregnancy at a younger age and those with multiple pregnancies exhibited lower adherence to healthy behaviors. This could be related to limited health knowledge, financial constraints, or competing responsibilities associated with caring for multiple children. Given these findings, interventions targeting younger and multiparous women should focus on improving their health awareness and access to resources that facilitate healthier lifestyle choices.

The mean FLRAS score of around 80 suggests that while pregnant women typically have a favorable attitude toward reading food labels, with a maximum score of 100, there is still room for improvement, indicating that some participants may not utilize them fully. Although no comparable studies using the FLRAS were found, it has been reported that 68.6% of pregnant women in the United States use food labels (Kim et al. 2017). This finding emphasizes the need for more education and strategies to improve label readability and increase knowledge of their importance in making informed food decisions (Viola et al. 2016). In this context, our study demonstrated a significant relationship between food label reading attitudes and healthy lifestyle behaviors, suggesting that using food labels plays a role in making informed dietary choices. However, there is a lack of direct studies investigating the influence of food labels on pregnant women. Nevertheless, studies conducted in different populations, including the elderly and younger, have observed that reading food labels is associated with healthier eating behaviors (Graham and Laska 2012; Kemaloglu and Kemaloğlu 2024; Verissimo et al. 2019). Moreover, a systematic review has suggested that the use of food labels may be linked to healthier dietary choices, potentially by reducing the consumption of nutrients of concern (energy, fat, and sodium), limiting unhealthy food options (salty snacks and desserts), and promoting the intake of beneficial nutrients and foods (fiber, protein, fruits, and vegetables) (Anastasiou et al. 2019). However, while food label reading contributes to dietary awareness, its impact on behavior change may be limited if individuals lack the necessary knowledge to interpret the information effectively (Giró‐Candanedo et al. 2022). Inadequate interpretive ability may prevent individuals from distinguishing between healthier and less healthy options, thereby reducing the potential of food labels to guide meaningful dietary changes. This highlights the importance of nutrition education programs that enhance label comprehension skills, particularly among vulnerable groups such as pregnant women. Future research should explore whether food label reading attitudes can impact healthy dietary behavior among pregnant women.

Food and nutrition literacy is crucial during pregnancy, enabling pregnant women to make informed nutrition decisions (Papežová et al. 2023). In this study, the total FNLS score ranged from 0 to 128, and the mean score of participants was 88.9. The FNLS consists of subscales with distinct scoring ranges, including knowledge (0–13), attitude (13–65), and behavior (10–50). The knowledge subscale score (8.3 out of 13) reflects a reasonable level of dietary knowledge, but some gaps need to be addressed. Jordanian pregnant women showed good nutritional knowledge, averaging 14.6 out of 20 (Abu‐Baker et al. 2021). In contrast, many studies, such as those conducted in Australia (mean score of 34.5 out of 76), Ethiopia (mean score of 4.1 out of 8), China (mean score of 9.5 out of 22) and the USA (mean score of 30 out of 57), reported lower levels of knowledge (Blondin and LoGiudice 2018; Lee et al. 2018; Wang et al. 2023; Yalewdeg et al. 2020). However, direct comparisons with previous studies are challenging due to differences in assessment tools and cut‐off points. Standardized approaches to evaluate food and nutrition knowledge in pregnant women are required. Furthermore, the mean attitude subscale score (50.5 out of 65) suggests a positive perspective on nutrition, which is an encouraging sign for health promotion efforts in the present study. Nonetheless, a good attitude toward nutrition is not always associated with high levels of knowledge or behavioral improvements. Despite their positive attitudes, pregnant women in this study were moderately committed to healthy nutrition behaviors, as evidenced by the behavior subscale score (30.1 out of 50). Similarly, Wang et al. (2023) have also shown that there was a gap between knowledge, attitude, and practice. This means that while there is an awareness and favorable attitude toward nutrition, external variables such as economic restrictions, accessibility, and cultural influences may limit attitude translation into actual behaviors. It is suggested that nutrition literacy is an essential component of healthy lifestyle behaviors, especially during pregnancy (Papežová et al. 2023). Our results confirmed this hypothesis by demonstrating that expecting mothers with higher FNLS scores are likelier to engage in healthier lifestyle behaviors. Several studies have highlighted that non‐adherence to pregnancy‐specific nutritional recommendations was linked with lower levels of nutrition knowledge (Aktaç et al. 2018; Bookari et al. 2016a; Lee et al. 2018) while indicating that, according to the cited studies, nutrition education during pregnancy was associated with better pregnancy outcomes (Aktaç et al. 2018). Mitran et al. (2024) have also emphasized that the nutrition literacy of mothers establishes the groundwork for promoting a favorable food environment in their future families and impacting the health behaviors of future generations. Despite these findings, there is currently a scarcity of research that directly examines the effect of nutrition literacy in altering dietary behaviors. Future studies should look into how nutrition literacy interventions influence maternal nutrition and dietary habits.

The regression analyses revealed that both food label reading attitudes and food and nutrition literacy were statistically significant predictors of healthy lifestyle behaviors, with food and nutrition literacy emerging as the stronger predictor (*β* = 0.21 vs. *β* = 0.15). These findings are consistent with Kempen et al. (2012), who similarly reported that food label reading practices were positively associated with healthier lifestyle behaviors, though the magnitude of the association was modest. The relatively small effect sizes observed in the present study (*R* ^2^ = 0.02–0.06) may be attributable to the multifactorial nature of lifestyle behavior during pregnancy, wherein psychosocial, cultural, and economic factors play substantial roles beyond literacy related variables alone. Notably, when both predictors were entered simultaneously, food label reading attitude lost independent significance while food and nutrition literacy remained a strong predictor, suggesting that the effect of label reading may be partly mediated by broader literacy competencies. This finding is not surprising given that Giró‐Candanedo et al. (2022) have pointed out that simply reading food labels may not translate into behavioral change unless individuals also possess the knowledge and skills to make sense of that information which is, at its core, what nutrition literacy entails. The similarities observed between our findings and those of previous studies may reflect shared determinants of nutrition‐related behaviors, such as the universal role of health literacy in guiding food choices during pregnancy. Conversely, the differences in effect sizes across studies may stem from variations in sample characteristics, cultural contexts, and the specific measurement tools employed, all of which can influence the strength of associations between literacy‐related variables and lifestyle behaviors.

Overall, while food label reading attitudes and nutrition literacy were statistically significant predictors of healthy lifestyle behaviors, the effect sizes were relatively modest. The confidence intervals indicated that each unit increase in these predictors was associated with only small improvements in lifestyle behaviors. This highlights that statistical significance does not necessarily translate into strong practical significance, emphasizing the need for broader educational, social, and policy‐level strategies to achieve meaningful behavioral change among pregnant women.

Despite its valuable contributions, this study has some limitations. First, its cross‐sectional design prevents us from establishing causal relationships between food label reading attitudes, nutrition literacy, and healthy lifestyle behaviors. The lack of longitudinal analysis further limits our ability to determine whether improvements in these areas translate to sustained behavioral changes over time. Second, data were collected through face‐to‐face interviews in which participants provided self‐reported answers. This method may introduce bias because participants may overestimate their adherence to healthy behaviors. In addition, social desirability bias cannot be excluded, as pregnant women may have provided responses that they perceived as more acceptable. Finally, the sample size is relatively limited, which may restrict the generalizability of the results. Future research with larger sample sizes and longitudinal designs should assess the long‐term impact of food label reading and nutrition literacy interventions.

## 5 Conclusion

This study underscores the importance of food label reading attitudes and nutrition literacy in promoting healthy lifestyle behaviors among pregnant women. Women with higher nutrition literacy and better food label reading skills are more likely to adopt healthier lifestyles. Based on the findings of this study, several recommendations can be made to promote healthy lifestyle behaviors among pregnant women. Firstly, public health interventions should focus on educational programs to improve nutrition literacy, particularly for expectant mothers. Additionally, healthcare providers should emphasize the importance of food label reading during regular prenatal visits, offering practical tips on effectively interpreting nutritional information. It is also recommended that targeted campaigns or workshops be developed to promote understanding of food labels, making this information more accessible and engaging. We lastly suggest that future research should investigate the effectiveness of interventions to improve food label reading skills and nutrition literacy among pregnant women. Examining the long‐term impact of such initiatives on maternal and neonatal health outcomes could provide valuable insights for public health strategies. Finally, these efforts should be integrated within routine prenatal care services to ensure consistent support and maximize the impact of nutrition literacy interventions.

## Author Contributions

**Aybike Gizem Köse:** data curation, formal analysis, visualization, writing – original draft, writing – review and editing. **Ozge Yesildemir:** supervision, writing – original draft, writing – review and editing. **Zehra Tuncay:** investigation, data curation, writing – original draft. **İrem İlayda Sezen:** investigation, data curation, writing – original draft. **Mehmet Arif Icer:** conceptualization, methodology, investigation, software, validation, data curation, supervision, writing – original draft, writing – review and editing.

## Funding

The authors thank Türkiye Bilimsel ve Teknolojik Araştırma Kurumu (TUBITAK) for providing the open‐access publication fee.

## Ethics Statement

The study protocol was approved by the Ethics Committee of Amasya University (protocol code: 2024/127; date of approval: 26 November, 2024). All participants provided informed written consent, and the study was conducted in adherence to the principles outlined in the Helsinki Declaration.

## Conflicts of Interest

The authors declare no conflicts of interest.

## Data Availability

The data that support the findings of this study are available on request from the corresponding author. The data are not publicly available due to privacy or ethical restrictions.
